# Propolis and it's emerging anti-neoplastic effects: beyond its role in oral dysplasia

**DOI:** 10.5935/1808-8694.20130074

**Published:** 2015-10-08

**Authors:** Shailendra Kapoor

**Affiliations:** a MD.

**Keywords:** cancer symptoms, cancer vaccines, cancerinism, propolis

## TO THE EDITOR

I read with great interest the recent article of Cavalcante et al.^1^. Propolis may exert a number of anti-neoplastic effects besides having an inhibitory effect on oral epithelial dysplasia.

For instance, growth inhibition is seen in breast cancer cell lines following administration of caffeic acid phenethyl ester (CAPE) derived from propolis. CAPE modulates intra-tumor angiogenesis as well causes tumoral apoptosis thus decreasing tumor growth in breast carcinomas^2^. It also attenuates tumor resistance to chemo-therapeutic agents by decreasing expression of the mdr-1 gene. Besides this, it also alters NF-κB function. CAPE treatment of breast cancer stem cells attenuates CD44 levels by almost 95%^3^. Thus, CAPE decreases self-renewal in breast carcinoma stem cells.

Similarly, methanolic extracts of Mexican propolis have recently been shown to exert cytotoxic effects against pancreatic cancer cell lines. These anti-proliferative effects in pancreatic cells are primarily exerted by two new phenylallylflavanones, (2R,3R)-6-[1-(4’-hydroxy-3’–methoxyphenyl)prop-2-en-1-yl]pinobanksin (1) and (2R,3R)-6-[1-(4’-hydroxy-3’-methoxyphenyl)prop-2-en-1-yl] pinobanksin 3-acetate (2)^4^.

Similarly, water extracts of Turkish propolis decrease cell viability to almost 18% when it is administered to prostate carcinoma cell lines^5^. Similarly, sensitivity to TRAIL induced apoptosis in prostate carcinoma cell lines is augmented by ethanol extracts of Brazilian green propolis^6^. Propolis modulates NF-κB function and thereby helps to overcome resistance to TRAIL. Quercetin, p-coumaric acid and artepillin C are the primary phenolic components that are responsible for these anti-neoplastic effects.

Similarly, Portuguese propolis exhibits cytotoxic effects against renal cell carcinoma cells. Similarly, red propolis inhibits growth in leukemia cell lines. The apoptotic capacity of red propolis is almost the same as gleevec. In addition, green propolis exerts anti-leukemic effects though it is not as potent as red propolis.

The above examples clearly illustrate the anti-neoplastic effects of propolis and the need for further studies in this regard.
